# Protective Effects of Estradiol on Disease Progression in a Murine Model of Fuchs Endothelial Corneal Dystrophy

**DOI:** 10.1167/iovs.66.15.64

**Published:** 2025-12-22

**Authors:** Itsuki Oka, Sohya Fujimoto, Tomohiro Fukui, Kota Mayumi, Theofilos Tourtas, Ursula Schlötzer-Schrehardt, Friedrich Kruse, Albert S. Jun, Noriko Koizumi, Naoki Okumura

**Affiliations:** 1 Department of Biomedical Engineering, Faculty of Life and Medical Sciences, Doshisha University, Kyotanabe, Japan; 2 Department of Ophthalmology, University of Erlangen-Nürnberg, Erlangen, Germany; 3 Department of Ophthalmology, University of Virginia School of Medicine, Charlottesville, Virginia, United States

**Keywords:** Fuchs endothelial dystrophy (FECD), transforming growth factor-beta (TGF-β), estrogen, estradiol, mouse model

## Abstract

**Purpose:**

The purpose of this study was to investigate the protective effects of estradiol (E2) on disease progression in Fuchs endothelial corneal dystrophy (FECD) and to explore potential underlying mechanisms.

**Methods:**

E2-supplemented drinking water was administered to *Col8a2*^Q455K/Q455K^ mice, a mouse model of FECD. The corneal endothelial phenotype was evaluated using contact specular microscopy. In vitro studies were performed using immortalized FECD (iFECD) cells derived from patients with and without TCF4 triplet repeat expansion to assess the effects of E2 on extracellular matrix (ECM) production and protein aggregation through immunofluorescence, whereas transforming growth factor-beta (TGF-β) signaling and epithelial-mesenchymal transition (EMT)-related factors were evaluated by Western blot analysis.

**Results:**

E2 treatment significantly reduced guttae formation (0.55 ± 0.23% vs. 0.97 ± 0.22%, *P* < 0.001) and maintained higher endothelial cell density (2263 ± 177 vs. 2058 ± 118 cells/mm², *P* = 0.004) in FECD mice compared with untreated controls at 20 weeks of age. In vitro studies demonstrated that E2 suppressed TGF-β2-induced upregulation of ECM proteins (fibronectin, biglycan, and collagen I) and reduced protein aggregation in both iFECD cell lines. Mechanistically, E2 inhibited TGF-β signaling by suppressing Smad2/3 phosphorylation and downregulating EMT-related factors (Snail and ZEB1).

**Conclusions:**

E2 ameliorates FECD progression by suppressing excessive ECM production. Our in vitro data suggest this protective effect is mediated through the inhibition of the TGF-β-Smad signaling pathway. These findings provide critical in vivo evidence for the therapeutic potential of E2, establishing a strong rationale for its clinical investigation as a novel treatment for FECD.

Fuchs endothelial corneal dystrophy (FECD) is a bilateral corneal disease characterized by the formation of extracellular matrix (ECM) excrescences, called guttae, that develop primarily in the central cornea.^1^ FECD progression is marked by increasing guttae density, which causes light scattering and subsequent visual impairment.^1^^–^^3^ The condition is accompanied by progressive corneal endothelial dysfunction, ultimately leading to corneal edema in advanced stages, accompanied by significant visual disability.^1^^–^^3^ FECD represents the most prevalent form of corneal dystrophy, with an estimated prevalence of 7.33% in adults.^4^ This high prevalence has established FECD as the leading indication for corneal transplantation worldwide.^5^

Whereas FECD has long been recognized as having a strong hereditary component, as evidenced by familial cases,^6^ the occurrence of sporadic cases suggests that both genetic and environmental factors contribute to its development.^1^^,^^7^^,^^8^ A striking epidemiological feature of FECD is its pronounced sex disparity, with prevalence being 1.66-fold to 3.50-fold higher in female patients than in male patients.^1^^,^^4^^,^^9^ Although the increased incidence after menopause suggests a potential regulatory role of sex hormones, the underlying mechanisms for this female predominance remain incompletely understood.^10^^–^^13^

A recent epidemiological study suggests that estrogen deficiency in postmenopausal women is a critical risk factor for FECD,^13^ highlighting the potential cytoprotective role of its major biologically active form, estradiol (E2). Indeed, E2 is known to counteract key pathological processes highly relevant to FECD; in other fibrotic disorders, it suppresses transforming growth factor-beta (TGF-β) signaling and the epithelial-mesenchymal transition (EMT)—pathways central to the excessive ECM production and cell loss that drive the disease.^14^^–^^18^ These converging lines of evidence led us to hypothesize that E2 supplementation could protect corneal endothelial cells from pathological remodeling and degeneration. Therefore, this study was designed to investigate the effects of E2 administration in a murine model of FECD and to explore the underlying molecular mechanisms using in vitro systems derived from patient tissues.

## Materials and Methods

### Ethics Statement

The present investigation adhered to the ethical guidelines established in the Declaration of Helsinki. The research protocol received institutional approval from both the Ethics Committee of Friedrich-Alexander University Erlangen-Nürnberg (FAU; reference number: 140_20 B) and the Ethics Committee for Scientific Research at Doshisha University (reference number: 20009). Donor Descemet membranes containing corneal endothelial cells (CECs) were harvested from individuals diagnosed with FECD who underwent Descemet membrane endothelial keratoplasty (DMEK) at the University of Erlangen-Nürnberg. Each participant provided written informed consent before tissue procurement.

### Animal Studies

All experimental procedures involving animals were performed in accordance with the ARVO Statement for the Use of Animals in Ophthalmic and Vision Research under protocols authorized by the Animal Care and Use Committee of Doshisha University (reference number: Doshisha-A-24085, Kyoto, Japan). Wild-type C57BL/6J mice were purchased from CLEA Japan, Inc. (Tokyo, Japan). The experimental FECD model mice harbor a homozygous *Col8a2* Q455K mutation (*Col8a2*^Q455K/Q455K^), as previously established (by author A.S.J.).^19^

### E2 Administration

β-Estradiol (E8875; Sigma-Aldrich, Burlington, MA, USA) was initially dissolved in 95% ethanol to prepare a stock solution at a concentration of 5 mg/mL.^20^ The stock solution was subsequently diluted with water to achieve a final E2 concentration of 4 µg/mL in 0.1% ethanol. This concentration was chosen based on previous reports using the same dosage in drinking water, where it was shown to be safe and effective in long-term studies.^20^ Given the technical challenges of performing wide-field corneal imaging and the ethical imperative to minimize unnecessary animal use, we selected this single, well-established dose to ensure safety and reproducibility while avoiding excessive animal consumption. Twelve *Col8a2*^Q455K/Q455K^ mice were randomly assigned to receive E2-supplemented drinking water starting at 8 weeks of age, with ad libitum access. The control group comprised 11 *Col8a2*^Q455K/Q455K^ mice that were provided drinking water containing 0.1% ethanol, also beginning at 8 weeks of age with ad libitum access. E2 administration was initiated at 8 weeks of age to avoid interfering with the hormonal regulation associated with sexual maturation, which typically occurs around this time point in mice.

Given that excessive exposure to female sex hormones has been reported to cause adverse effects, including abdominal distension, urinary retention, and mortality,^21^ the mice were regularly monitored through physical examination and body weight measurements. No adverse effects were observed in any of the E2-treated animals throughout the study period.

### Corneal Endothelial Assessment by Contact Specular Microscopy

The investigation comprised 3 experimental cohorts evaluated at 20 weeks of age: FECD model mice (*Col8a2*^Q455K/Q455K^, *n* = 11; one mouse was lost during the observation period), E2-treated FECD model mice (*Col8a2*^Q455K/Q455K^, *n* = 12), and age-matched wild-type controls (C57BL/6J mice provided standard drinking water, *n* = 9).

For endothelial imaging, the mice were subjected to general anesthesia, followed by administration of Benoxyl ophthalmic solution (2 µL; Santen Pharmaceutical, Osaka, Japan) and Scopisol (Senju Pharmaceutical, Osaka, Japan) to the right cornea. The corneal endothelium was visualized using a contact specular microscope (Konan Medical, Hyogo, Japan). Endothelial cell density (ECD) was quantitatively assessed based on 3 images selected at random from a pool of 100 video-extracted still frames. Masked observers conducted the analysis using a previously validated U-Net convolutional neural network algorithm designed for cell skeleton delineation.^22^ For comprehensive visualization, panoramic images were generated following our established protocol. The methodology involved selecting optimally focused frames at 0.2-second intervals based on normalized roughness coefficients, yielding 400 grayscale images (480 × 720 pixels).^22^ Following Laplacian transformation-based quality selection of 100 frames, panoramic reconstruction was accomplished using AutoStitch software.^23^ Subsequently, guttae were identified through deep learning algorithms, enabling the calculation of the guttae-to-corneal area ratio.

### Establishment of FECD Cell Lines

Human corneal endothelial cells (HCECs) were obtained from the Descemet membranes of two patients with FECD who were stratified based on their *TCF4* CTG repeat status (≥50 or <50 repeats) via genomic DNA analysis.^7^^,^^24^ Following established protocols,^25^^,^^26^ primary HCECs were extracted by enzymatic treatment using collagenase A (1 mg/mL; Roche Applied Science, Penzberg, Germany) in OptiMEM-I (Life Technologies Corp., Carlsbad, CA, USA) at 37°C for 16 hours. The harvested HCECs were plated onto surfaces coated with laminin E8 fragments (iMatrix-511; Nippi, Inc., Tokyo, Japan) and maintained in OptiMEM-I enriched with 8% FBS, Y-27632 (10 µM; Wako Pure Chemical Industries, Ltd., Osaka, Japan; initial 24 hours only), SB203580 (10 µM; Wako Pure Chemical Industries, Ltd.), SB431542 (1 µM; Wako Pure Chemical Industries, Ltd.), epidermal growth factor (5 ng/mL; Life Technologies Corp.), ascorbic acid (20 µg/mL; Sigma-Aldrich), calcium chloride (200 mg/L; Sigma-Aldrich), chondroitin sulfate (0.08%; Sigma-Aldrich), and gentamicin (50 µg/mL; Life Technologies Corp.). The cells were cultured at 37°C in 5% CO_2_, with fresh medium provided every 2 days.

Following 14 to 21 days of culture, the primary cells were immortalized by lentiviral delivery of SV40 large T antigen and hTERT genes. Lentiviral constructs were prepared via polymerase chain reaction (PCR) amplification and TA-cloning, followed by cotransfection with helper plasmids into HEK293T cells (RCB2202; Riken Bioresource Center, Ibaraki, Japan). Viral preparations were collected after 48 hours and applied to the HCECs with polybrene (5 µg/mL). The established cell lines, termed iFECD (RE+) and iFECD (RE−) according to their CTG repeat profiles, were propagated in Dulbecco's modified Eagle's medium (DMEM; Life Technologies Corp.) containing 10% fetal bovine serum (FBS) and 1% penicillin/streptomycin (Nacalai Tesque, Kyoto, Japan). Subculturing was performed using 0.05% trypsin–ethylene diamine tetraacetic acid (EDTA; Life Technologies Corp.) when the cultures reached 80% confluence.

The iFECD (RE+) and iFECD (RE−) cells were cultured until confluent and further cultured with fresh DMEM supplemented with TGF-β (10 ng/mL; Wako Pure Chemical Industries, Ltd.) for 24 hours for some experiments. This concentration was selected based on our previous studies using the same model, to ensure consistency with our established experimental conditions.^27^^,^^28^ To evaluate cytotoxicity, iFECD (RE+) cells were exposed to either the dimethylsulfoxide (DMSO) vehicle control (Nacalai Tesque) or E2 (100 nM, 1 µM, or 10 µM; Sigma-Aldrich) in DMEM supplemented with 2% FBS and 1% penicillin/streptomycin for 48 hours. Cell viability measurements were performed using the Cell Titer-Glo Luminescent Assay system (Promega, Madison, WI, USA). After removing the culture medium to achieve a final volume of 50 µL/well, 50 µL of Cell Titer-Glo reagent (Promega) was added to each well at a 1:1 ratio with the remaining medium. The plate was mixed on a shaker at approximately 120 rpm for 2 minutes and incubated at room temperature for 10 minutes to stabilize the luminescence signal. Subsequently, 50 µL of the reaction mixture was transferred to a white polystyrene 96-well assay plate, and luminescence was measured using the GloMax-Multi Detection System (Promega).

### Immunofluorescence and Aggresome Staining

For immunofluorescence staining, iFECD (RE+) or iFECD (RE−) cells were seeded onto circular glass coverslips (Matsunami Glass, Osaka, Japan) in 24-well plates at a density of 8 × 10^3^ cells/well. The cells were washed twice with calcium-free and magnesium-free phosphate-buffered saline (PBS) and fixed with 4% paraformaldehyde (PFA; Nacalai Tesque) for 10 minutes at room temperature. The cells were permeabilized with 0.5% Triton X-100 (Nacalai Tesque) and blocked with 2% bovine serum albumin (BSA; Nacalai Tesque). Primary antibodies included mouse anti-fibronectin (1:1000; BD Biosciences, San Jose, CA, USA), rabbit anti-biglycan (1:1000; Sigma-Aldrich), and rabbit anti-collagen I (1:200; RKL Rockland Immunochemicals, Limerick, PA, USA). Secondary antibodies comprised Alexa Fluor 488 goat anti-mouse IgG (H+L) and Alexa Fluor 488 donkey anti-rabbit IgG (H+L) (both 1:1000; Thermo Fisher Scientific Inc., Waltham, MA, USA). Nuclei were counterstained with 4′,6-diamidino-2-phenylindole (DAPI; 1:1000; Dojindo Laboratories, Kumamoto, Japan).

For protein aggregate detection, cells were incubated with Aggresome Detection Reagent (1:1000; Enzo Life Science Inc., Farmingdale, NY, USA) and DAPI (1:1000; Dojindo Laboratories) in calcium-free and magnesium-free PBS for 60 minutes at room temperature following PFA fixation and Triton X-100 permeabilization, as described above. Fluorescence images were acquired using a Leica DM 2500 microscope (Leica Microsystems, Wetzlar, Germany).

### Immunoblotting Analysis

Floating and dead cells were collected by harvesting the culture medium on ice, and adherent cells were washed twice with 1 × PBS (−). The wash solutions were combined, and the samples were centrifuged at 800×*g* for 10 minutes at 4°C. The supernatant was discarded, and the resulting pellet was retained. The washed adherent cells were lysed on ice using radioimmunoprecipitation assay buffer (RIPA; 50 mM Tris-HCl [pH 7.4], 150 mM NaCl, 1 mM EDTA, 0.1% SDS, 0.5% sodium deoxycholate, and 1% NP-40) for protein extraction. The pellet from the floating and dead cell fractions was also resuspended and included in the protein extraction process. Lysates were sonicated on ice using a BIORUPTOR (Tosho Denki, Tokyo, Japan) for three 30-second cycles in cold water, followed by centrifugation at 15,000 rpm for 10 minutes at 4°C. The supernatant containing proteins was collected for further analysis.

Proteins were separated by sodium dodecyl sulfate polyacrylamide gel electrophoresis (SDS-PAGE) and transferred to FluoroTrans W polyvinylidene fluoride (PVDF) transfer membranes (Pall Corporation, Port Washington, NY, USA). Primary antibodies included rabbit anti-Smad2 (1:1000), rabbit anti-phospho-Smad2 (1:1000), rabbit anti-Smad3 (1:1000), rabbit anti-phospho-Smad3 (1:1000), rabbit anti-snail (1:1000), and rabbit anti-ZEB1 (1:1000; all from Cell Signaling Technology, Danvers, MA, USA); mouse anti-fibronectin (1:15000; BD Biosciences, San Jose, CA, USA); rabbit anti-biglycan (1:1000; Sigma-Aldrich); and mouse anti-GAPDH (1:3000; Medical & Biological Laboratories, Nagoya, Japan). Horseradish peroxidase-conjugated anti-rabbit or anti-mouse secondary antibodies (1:5000; GE Healthcare, Chicago, IL, USA) were used for detection. All antibodies were diluted in 3% nonfat dry milk (Nacalai Tesque). Protein bands were visualized using Chemi Lumi ONE Ultra (Nacalai Tesque) and analyzed using an Image Quant LAS 4000mini system (Fujifilm, Tokyo, Japan).

To validate the in vitro findings in vivo, corneal endothelial tissues were collected from wild-type C57BL/6J mice, *Col8a2*^Q455K/Q455K^ mice (FECD model mice), and estradiol-treated FECD model mice at 28 weeks of age. Both corneas from each mouse were excised under a stereomicroscope, and the Descemet membrane–endothelial complex was isolated by gentle peeling using fine forceps. The tissues were pooled within each group (wild type, *n* = 10; FECD, *n* = 10; and FECD + E2, *n* = 9), immediately lysed in RIPA buffer supplemented with protease and phosphatase inhibitors, and analyzed by Western blotting, as described above using the same set of primary antibodies.

### Quantitative PCR Analysis

Total RNA was extracted from cells using the RNeasy Mini Kit (QIAGEN, Hilden, Germany) following the manufacturer’s protocol. Briefly, the culture medium was removed, and cells were washed twice with 1 × PBS (−). After the addition of 350 µL Buffer RLT per well, cells were scraped using a cell scraper. The lysate was mixed with 350 µL of 70% ethanol and transferred to a RNeasy Mini Spin Column, followed by centrifugation at 10,000×*g* for 15 seconds. The column was washed with 350 µL Buffer RW1, and DNA contamination was removed by adding a mixture of 70 µL Buffer RDD and 10 µL RQ1 RNase-Free DNase (Promega) and incubating at room temperature for 20 minutes. Subsequent washes with 350 µL Buffer RW1 and 500 µL Buffer RPE (repeated twice) were performed, followed by centrifugation at 14,000×*g* for 2 minutes. RNA was eluted with 50 µL RNase-free water and quantified using a NanoDrop spectrophotometer (Thermo Fisher Scientific Inc.).

The cDNA was synthesized using 100 ng of total RNA in a 20 µL reaction mixture containing 4 µL 5× RT Buffer, 2 µL 10 mM deoxynucleotide triphosphates (dNTPs), 1 µL 25 µM random primer (Thermo Fisher Scientific Inc.), 1 µL ReverTra Ace (TOYOBO, Osaka, Japan), and 1 µL RNase Inhibitor (TOYOBO). The reaction conditions were 30°C for 10 minutes (annealing), 42°C for 1 minute (extension), and 99°C for 5 minutes (denaturation).

Gene expression levels were analyzed using TaqMan real-time PCR (Applied Biosystems, Foster City, CA, USA). The TaqMan primers used were *GAPDH*, Hs00266705_mL; *FN1*, Hs00365052_mL; *BGN*, Hs00959143_mL; and *COL1A1*, Hs00164004_mL (Applied Biosystems). Quantitative real-time PCR was performed on a QuantStudio 3 Real-Time PCR System (Thermo Fisher Scientific Inc.) with the following cycling conditions: initial denaturation at 95°C for 20 seconds, followed by 40 cycles of 95°C for 1 second and 60°C for 20 seconds.

### Statistical Analysis

Areas of guttae and endothelial cell density are presented as box-and-whisker plots, where boxes represent the interquartile range (IQR), with horizontal lines indicating the median and “+” symbols showing the mean values. Whiskers extend to the minimum and maximum values. Between-group comparisons of contact specular microscope measurements were analyzed using Welch's *t*-test, whereas multiple group comparisons were performed using Dunnett’s test.

All statistical analyses were conducted using R (version 4.3.0). The multcomp library (version 1.4–25) was used for Dunnett's multiple-comparisons test. Statistical significance was defined as *P* < 0.05.

## Results

### Effects of E2 on the Corneal Endothelial Phenotype in a Mouse Model of FECD

**Figure 1.**
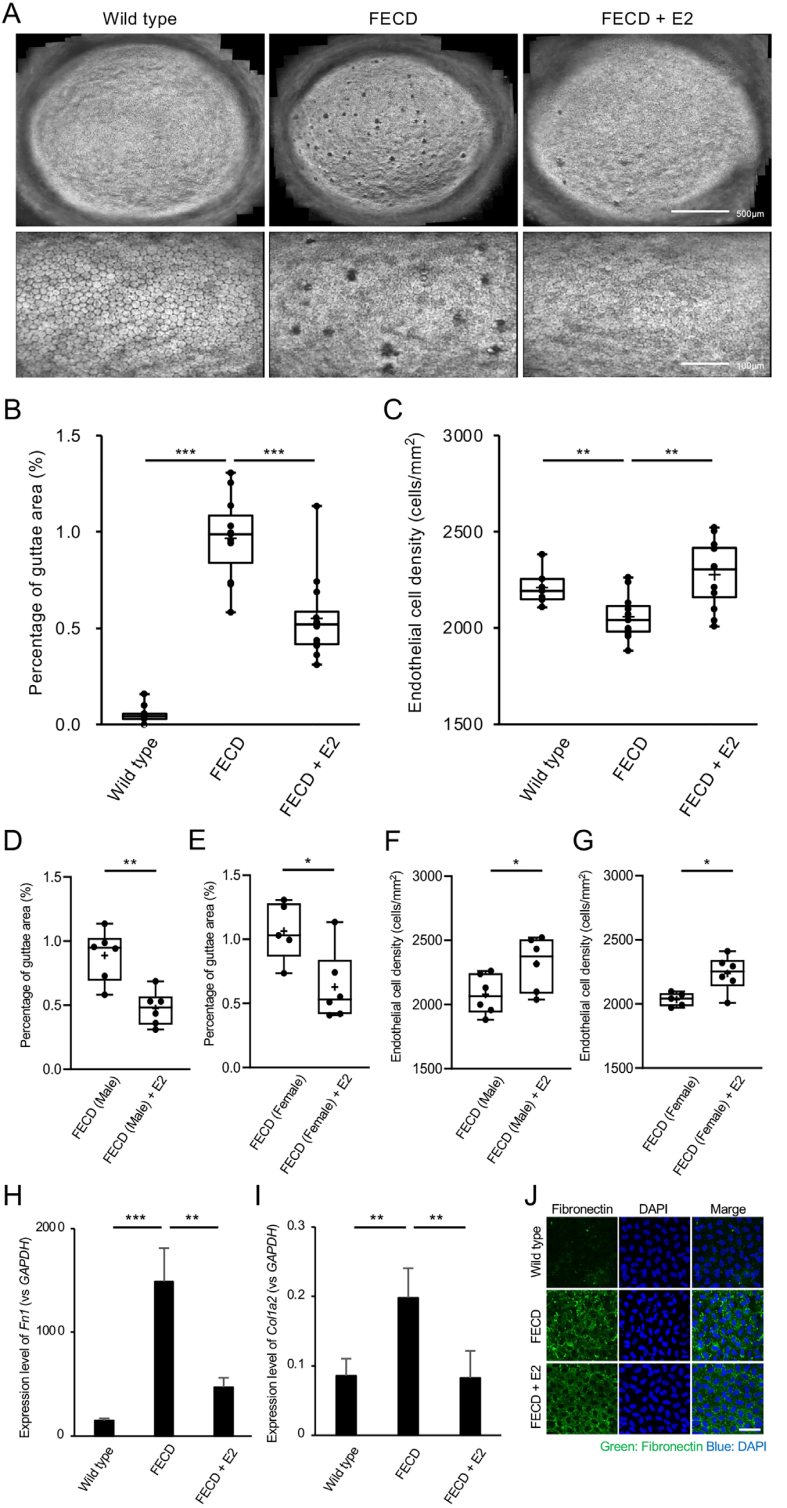
**In vivo estradiol (E2) treatment ameliorates the corneal endothelial phenotype in an FECD mouse model.** (**A**) Effects of E2 on the corneal endothelial phenotype were investigated in *Col8a2*^Q455K/Q455K^ mice, a mouse model of FECD, by supplying E2-supplemented drinking water ad libitum. Representative specular microscopy images of the corneal endothelium from the wild-type, FECD, and FECD + E2 groups. FECD mice exhibited extensive guttae formation and enlarged endothelial cells compared to wild-type mice. E2 treatment reduced guttae formation and maintained smaller endothelial cells with a higher cell density compared with untreated FECD mice. (**B**) Quantification of guttae formation in the wild-type (*n* = 9), FECD (*n* = 11), and FECD + E2 (*n* = 12) groups. The percentage of guttae area was significantly lower in the FECD + E2 group (0.55 ± 0.23%) compared with the FECD group (0.97 ± 0.22%, *P* < 0.001). (**C**) Quantification of endothelial cell density (ECD). The FECD + E2 group maintained a significantly higher endothelial cell density (2,263 ± 177 cells/mm²) compared with the FECD group (2058 ± 118 cells/mm²; *P* = 0.004). (**D, E**) A sex-stratified analysis shows that E2 administration significantly reduced the percentage of guttae area in both male mice (**D**; FECD, *n* = 6; FECD + E2, *n* = 6; *P* = 0.002) and female mice (**E**; FECD, *n* = 5; FECD + E2, *n* = 6; *P* = 0.018) compared with their respective untreated controls. (**F, G**) Similarly, the analysis of endothelial cell density, stratified by sex, shows that E2 treatment resulted in a significantly higher density in both male mice (**F**; FECD, *n* = 6; FECD + E2, *n* = 6; *P* = 0.049) and female mice (**G**; FECD, *n* = 5; FECD + E2, *n* = 6; *P* = 0.013). (**H, I**) Quantitative PCR analysis shows that the expression of the ECM-related genes FN1 (**H**) and COL1A1 (**I**), which was elevated in the FECD group, was significantly suppressed by E2 administration. (**J**) Immunofluorescence for fibronectin (*green*) revealed minimal deposition in wild-type corneas, whereas its accumulation was evident in the corneas of FECD model mice. This pathological deposition was markedly suppressed following E2 treatment. Nuclei were counterstained with DAPI (*blue*). Images are representative of four independent experiments. *Scale bar* = 100 µm.

The corneal endothelium was examined using a contact specular microscope in wild-type mice (wild-type group; *n* = 9), FECD model mice (FECD group; *n* = 11), and FECD model mice treated with E2 (FECD + E2 group; *n* = 12). At 20 weeks of age, the corneal endothelia of the wild-type group showed almost no guttae formation and maintained a monolayer of polygonal cells similar to that of healthy human corneal endothelium. In contrast, the FECD group exhibited phenotypic features of FECD, including guttae formation and enlarged endothelial cells with decreased cell density. The FECD + E2 group showed less guttae formation, less endothelial cell enlargement, and higher cell density compared with the FECD group (Fig. 1A).

At 20 weeks, the mean percentage of guttae was 0.053 ± 0.049% in the wild-type group, 0.97 ± 0.22% in the FECD group, and 0.55 ± 0.23% in the FECD + E2 group (Fig. 1B). Statistical analysis revealed significant differences between the FECD and FECD + E2 groups (*P* < 0.001), indicating that E2 treatment significantly reduced guttae formation in FECD mice. The mean endothelial cell density was 2211 ± 86 cells/mm² in wild-type mice, 2058 ± 118 cells/mm² in the FECD group, and 2263 ± 177 cells/mm² in the FECD + E2 group (Fig. 1C). Significant differences were observed between the FECD and FECD + E2 groups (*P* = 0.004), demonstrating that E2 treatment maintained significantly higher endothelial cell density in FECD mice. We also conducted sex-specific analyses to evaluate the effects of E2 administration. In male mice, the mean percentage of guttae area was significantly lower in the FECD + E2 group than in the untreated FECD group (*P* = 0.002; Fig. 1D). A similar significant reduction in guttae formation was observed in female mice following E2 administration (*P* = 0.018; Fig. 1E). Furthermore, endothelial cell density was significantly preserved in the FECD + E2 group compared with the FECD group in both male (*P* = 0.049; Fig. 1F) and female mice (*P* = 0.013; Fig. 1G).

To explore the molecular mechanisms underlying these improvements, it is important to consider the core pathology of FECD, which is the formation of guttae—excrescences of abnormally accumulated ECM. Key components of this pathological ECM include fibronectin and collagen I, and their deposition into guttae is the primary cause of the light scattering and subsequent visual impairment characteristic of the disease. We therefore analyzed the expression of ECM-related genes and proteins. Quantitative PCR showed that the expression of *FN1* (Fig. 1H) and *COL1A1* (Fig. 1I) was significantly elevated in the FECD group, whereas E2 administration significantly suppressed their expression (*P* = 0.002 for both). Immunofluorescence analysis of fibronectin (Fig. 1J) corroborated these results at the protein level, revealing increased accumulation in the FECD group and a marked reduction in the FECD + E2 group. These results demonstrate that E2 administration effectively mitigates ECM overproduction in FECD mice.

### Effects of E2 on Extracellular Matrix Production and Protein Aggregation in FECD Model Cells

**Figure 2.**
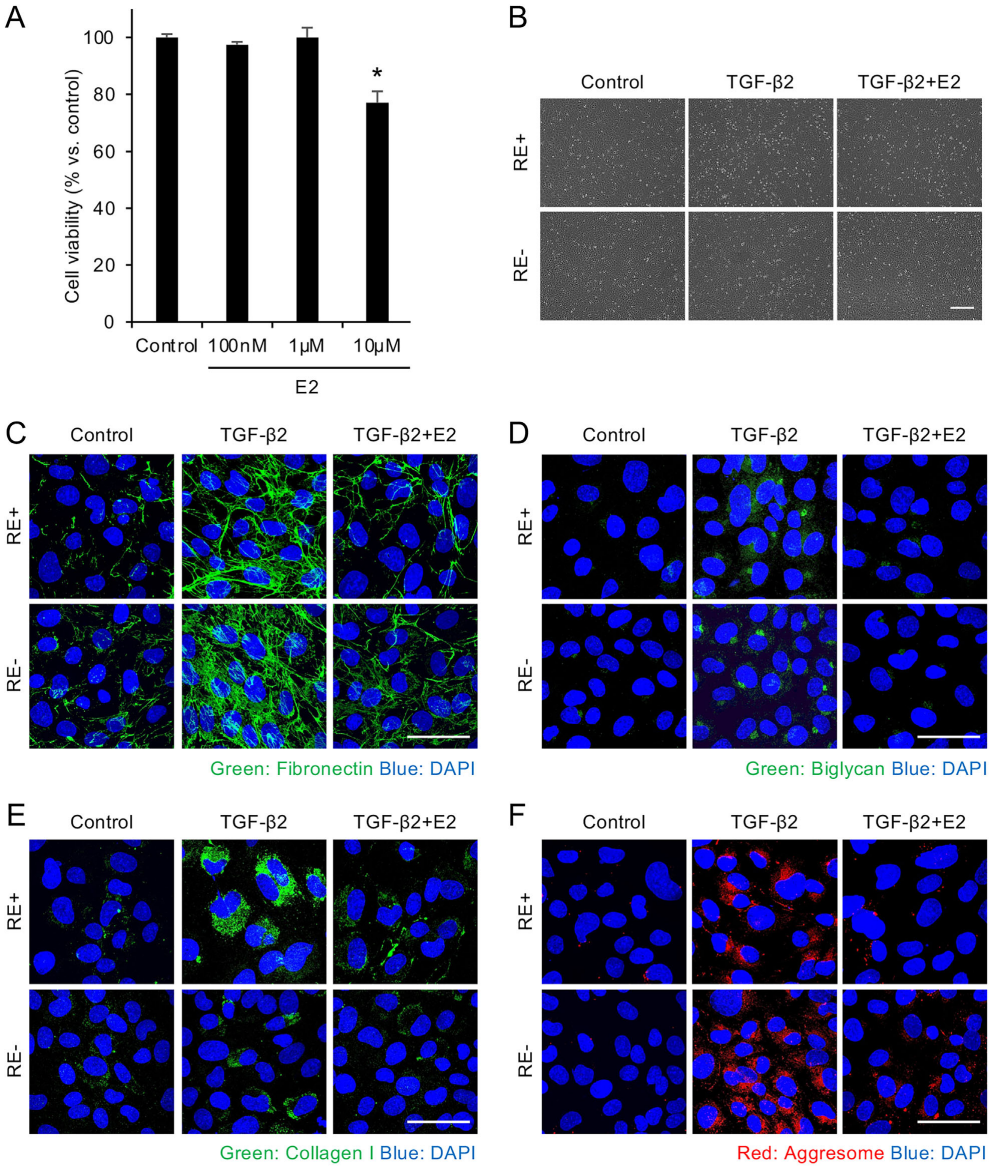
**Effects of estradiol (E2) on extracellular matrix production and protein aggregation in FECD model cells.** (**A**) Cell viability remained unchanged at E2 concentrations of 100 nM and 1 µM but decreased significantly at 10 µM E2 (*P < 0.05, *n* = 8; Dunnett’s multiple-comparisons test). (**B**) Representative phase-contrast microscopy images of disease model cells derived from patients with FECD with and without TCF4 triplet repeat expansion (iFECD (RE+) and iFECD (RE−), respectively). The cells were treated with TGF-β2 for 48 hours, followed by 24 hours of E2 treatment. No morphological changes were observed under any treatment conditions. *Scale bar* = 100 µm. (**C–E**) Immunofluorescence analysis of extracellular matrix proteins in iFECD cells. Cells were stimulated with TGF-β2 for 48 hours, followed by 24 hours of E2 treatment. TGF-β2 increased the expression of fibronectin (**C**), biglycan (**D**), and collagen I (**E**) in both cell lines, whereas co-treatment with E2 (1 µM) suppressed this upregulation. (**F**) Assessment of unfolded protein aggregation using aggresome staining. TGF-β2 stimulation increased aggresome formation (*red fluorescence*) in both cell lines, indicating enhanced protein aggregation. E2 cotreatment (1 µM) suppressed this TGF-β2-induced protein aggregation. Nuclei were counterstained with DAPI. *Scale bar* = 50 µm. Representative images from at least three independent experiments.

We investigated the in vitro effects of E2 on excessive ECM production in FECD cells utilizing disease model cells derived from patients with FECD with (iFECD (RE+)) and without (iFECD (RE−)) the *TCF4* triplet repeat expansion. Initially, we evaluated E2 cytotoxicity in the disease model cells (iFECD (RE+)) using the Cell Titer-Glo Luminescent Assay. However, cell viability significantly decreased at 10 µM E2; therefore, we conducted subsequent experiments at 1 µM E2, a concentration one-tenth the toxic dose, to ensure an adequate safety margin (Fig. 2A). This concentration was selected based on its widespread use in estrogen-related cell culture studies and its ability to elicit reproducible biological responses without toxicity.

Phase-contrast microscopy revealed no visible morphological changes in either iFECD (RE−) or iFECD (RE+) cells following treatment with TGF-β2 alone or in combination with E2 (Fig. 2B). However, immunofluorescence analysis demonstrated that TGF-β2 treatment increased the expression of fibronectin, biglycan, and collagen I in both cell lines compared with the controls. Co-treatment with E2 (1 µM) significantly suppressed this TGF-β2-induced upregulation of fibronectin (Fig. 2C), biglycan (Fig. 2D), and collagen I (Fig. 2E).

We then examined the effect of E2 on protein aggregation using aggresome staining in both cell lines. TGF-β2 treatment increased the accumulation of aggresomes (red fluorescence) compared with the controls, indicating enhanced protein aggregation. However, treatment with E2 significantly reduced this protein aggregation, demonstrating a protective effect of E2 against unfolded protein deposition in FECD model cells (Fig. 2F).

We further confirmed this inhibitory effect of E2 on ECM overproduction at the gene expression level (Supplementary Fig. S1). Quantitative PCR analysis revealed that TGF-β2 treatment significantly upregulated the expression of FN1, BGN, and COL1A1 in both cell lines compared with the controls. In the iFECD (RE+) cells, co-treatment with 1 µM E2 significantly suppressed the TGF-β2-induced upregulation of COL1A1 but showed no significant effect on FN1 and BGN expression. Notably, E2 co-treatment of the iFECD (RE−) cells significantly inhibited the TGF-β2-induced upregulation of all three genes: FN1, BGN, and COL1A1. Although differences in response patterns were observed between RE+ and RE– cells, the overall trend showed that E2 had a suppressive effect on ECM components at the gene expression level, corroborating our protein-level findings from immunofluorescence analysis.

### E2 Inhibits TGF-β Signaling Pathway in FECD

**Figure 3.**
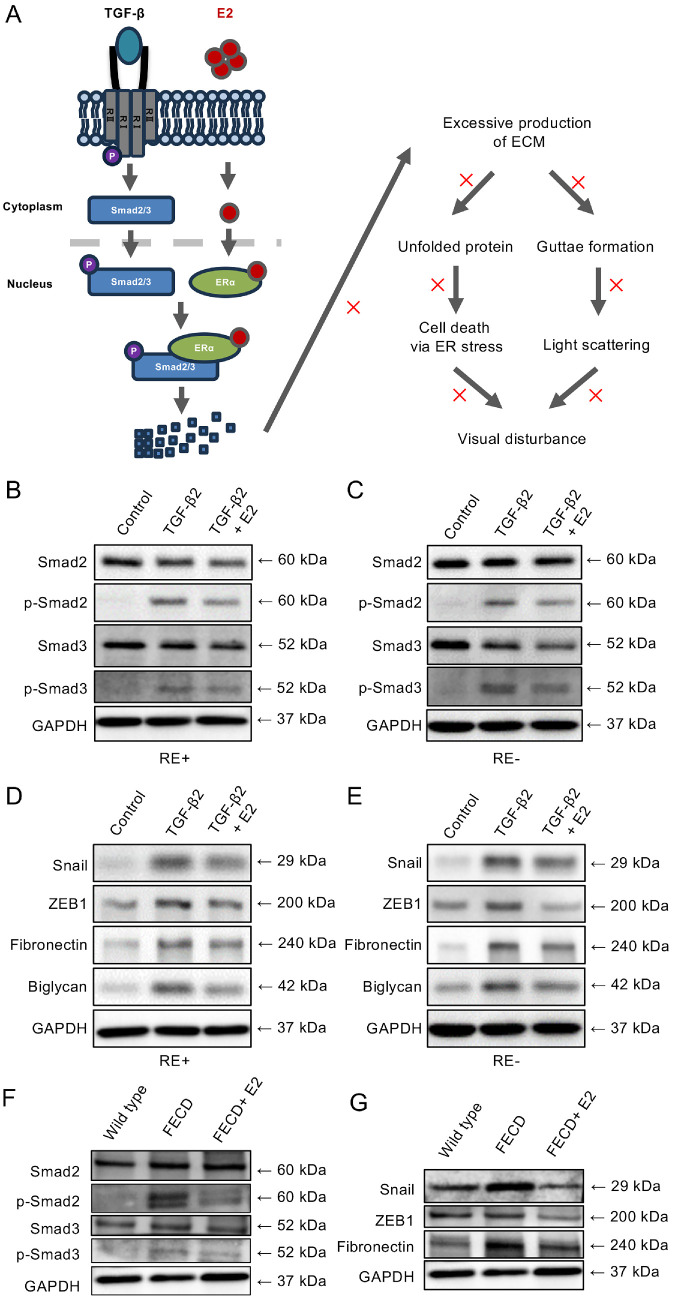
**In vitro estradiol (E2) inhibits the TGF-β signaling pathway in FECD.** (**A**) Schematic illustration of the proposed mechanism: E2 promotes the degradation of phosphorylated Smad proteins, inhibiting Smad-mediated signal transduction and suppressing TGF-β target genes, including those encoding extracellular matrix (ECM) components. (**B, C**) Western blot analysis was performed to examine Smad protein expression in immortalized FECD (iFECD) cells treated with TGF-β2 (10 ng/mL) for 48 hours, followed by E2 (1 µM) for an additional 24 hours. TGF-β2 treatment significantly increased phosphorylation of Smad2 and Smad3 compared to untreated controls. E2 treatment subsequently suppressed TGF-β2-induced Smad2/3 phosphorylation. (**D, E**) Expression of epithelial-mesenchymal transition (EMT) markers and ECM proteins was assessed by Western blot analysis in iFECD cells under the same treatment conditions. TGF-β2 stimulation upregulated the expression of EMT transcription factors Snail and ZEB1, along with ECM proteins fibronectin and biglycan. E2 treatment attenuated the TGF-β2-mediated induction of these proteins. Representative images from at least two independent experiments. (**F, G**) In vivo validation of E2-mediated inhibition of the TGF-β/Smad signaling pathway. Western blot analysis of corneal endothelium from wild-type (WT), FECD, and E2-treated FECD (FECD + E2) mice. (F) Phosphorylation status of Smad2 and Smad3: FECD mice exhibited marked elevation of phosphorylated Smad2/3 compared with WT controls, confirming activation of the canonical TGF-β/Smad pathway. E2 treatment attenuated this phosphorylation while leaving total Smad levels unchanged. Densitometric analysis (p-Smad/Smad, FECD set = 1.00) showed relative values of 0.09 (WT), 1.00 (FECD), and 0.26 (FECD + E2) for p-Smad2/Smad2, and 0.16, 1.00, and 0.28 for p-Smad3/Smad3. (**G**) Expression analysis of EMT-associated transcription factors and ECM components: Snail, ZEB1, and fibronectin were upregulated in FECD mice but reduced following E2 treatment. Normalized expression values (relative to FECD = 1.00) were 0.42, 0.52, and 0.15 in WT; 1.00, 1.00, and 1.00 in FECD; and 0.27, 0.16, and 0.33 in FECD + E2, respectively. All protein intensities were normalized to total Smad (for phosphorylated forms) or GAPDH (for Snail, ZEB1, and fibronectin). Representative data from at least two independent analyses.

Previous studies have demonstrated that E2 suppresses TGF-β-induced cellular damage, such as excessive ECM production, by inhibiting Smad signaling in fibrosis and breast cancer models.^14^^–^^18^ Therefore, we hypothesized that a similar mechanism might exist in FECD (Figs. 3A). Western blot analysis revealed that TGF-β2 treatment increased the expression of phosphorylated Smad2 and Smad3 in both iFECD (RE−) and iFECD (RE+) cells compared with controls, but co-treatment with E2 significantly suppressed this TGF-β2-induced phosphorylation of Smad2 and Smad3 (Figs. 3B, 3C). Densitometric analysis confirmed a significant E2-mediated inhibition of Smad2 and Smad3 phosphorylation in both iFECD (RE+) (Supplementary Figs. S2A, S2B) and iFECD (RE−) cells (Supplementary Figs. S2C, S2D).

Further investigation of the effects of E2 on EMT-related factors and ECM-related proteins using Western blot analysis revealed that TGF-β2 treatment upregulated the expression of EMT-related factors (Snail and ZEB1) and ECM-related proteins (fibronectin and biglycan) in both cell lines compared with controls, but E2 co-treatment effectively suppressed this TGF-β2-induced upregulation of these proteins (Figs. 3D, 3E). Densitometric quantification confirmed that E2 significantly reduced the TGF-β2-induced expression of Snail and ZEB1 in both iFECD (RE+; see Supplementary Figs. S2E, S2F) and iFECD (RE−; cells (Supplementary Figs. S2G, S2H).

Similarly, E2 significantly suppressed the TGF-β2-induced expression of fibronectin and biglycan in both iFECD (RE+; Supplementary Figs. S2I, S2J) and iFECD (RE−) cells (Supplementary Figs. S2K, S2L). These results suggest that E2 inhibits the EMT and subsequent excessive ECM production in FECD model cells by suppressing the activation of the TGF-β/Smad signaling pathway.

To validate whether this inhibitory effect on the TGF-β/Smad signaling cascade occurs in vivo, we analyzed corneal endothelial tissues from wild-type, FECD, and E2-treated FECD mice. Western blot analysis revealed that phosphorylation of both Smad2 and Smad3 was elevated in FECD mice compared with wild-type controls, confirming activation of the canonical TGF-β/Smad pathway. Remarkably, E2 administration attenuated this phosphorylation without affecting total Smad protein levels (Fig. 3F), demonstrating that E2 effectively suppresses Smad activation in vivo. We further investigated the downstream consequences of this pathway modulation by examining EMT-associated transcription factors and ECM components. FECD mice displayed pronounced upregulation of Snail and ZEB1, accompanied by increased fibronectin deposition—hallmarks of active EMT and pathological ECM remodeling. Importantly, E2 treatment significantly mitigated these alterations, resulting in reduced expression of Snail, ZEB1, and fibronectin compared with untreated FECD mice (Fig. 3G).

## Discussion

In this study, we demonstrated that the administration of E2 in drinking water to our FECD mouse model suppressed two hallmark phenotypes of FECD: guttae formation and decreased corneal endothelial cell density. Further in vitro studies on disease model cells established from patients with FECD revealed that E2 inhibits excessive ECM production by suppressing the activation of the Smad signaling pathway. These findings strongly support our guiding hypothesis—formed from both clinical and mechanistic insights—that estrogen deficiency contributes to FECD progression, and that E2 exerts a cytoprotective effect by modulating TGF-β–driven fibrosis. The significance of this E2-mediated suppression of ECM is substantial, as its pathological accumulation manifests as both guttae and the vision-impairing posterior fibrillar layer (PFL) in FECD.

FECD progression is primarily characterized by guttae formation, and current clinical grading systems are based on the extent and density of guttae.^6^^,^^29^ However, as the disease advances beyond the formation of confluent guttae, a condition known as “buried guttae” develops, where dense ECM deposits form a PFL that covers the guttae.^30^^–^^33^ At this stage, many patients experience visual impairment due to light scattering and require corneal transplantation procedures, such as DMEK, or removal of guttae and PFL through Descemet's stripping only (DSO; a more recently developed procedure).^2^^,^^3^ This clinical trajectory thus underscores that aberrant ECM accumulation is the central driver of both structural and functional decline of the corneal endothelium. In this context, our observation that E2 attenuates ECM overproduction—potentially through modulation of the TGF-β/Smad signaling cascade—suggests a novel therapeutic approach for addressing this key pathogenic mechanism in FECD. Notably, this mechanistic effect was reproducible across both cellular and animal models. In iFECD cells, E2 treatment suppressed TGF-β2–induced phosphorylation of Smad2/3 and reduced the expression of EMT-associated transcription factors and ECM proteins. Correspondingly, in vivo analysis of corneal tissues from FECD model mice revealed a similar response: E2 administration attenuated Smad2/3 phosphorylation and diminished the expression of Snail, ZEB1, and fibronectin. The consistency between these in vitro and in vivo findings supports the hypothesis that E2 may ameliorate FECD pathology by modulating the TGF-β/Smad–EMT–ECM axis in corneal endothelial cells.

It is important to reconcile our results with a recent study by Kumar and colleagues,^12^ which reported that estrogen metabolites—particularly those produced via CYP1B1 under oxidative stress—can be genotoxic to corneal endothelial cells. This apparent discrepancy, however, likely reflects distinct experimental contexts rather than a direct contradiction. Our study administered systemic E2 in the absence of exogenous oxidative stress, a condition that may have favored the emergence of its protective effects. This is consistent with the well-documented dual nature of estrogen, which can act as either a cytoprotective or a cytotoxic agent depending on factors such as dose, local redox status, and metabolic pathways.^34^ Therefore, our work and that of Jurkunas et al. likely provide complementary insights, collectively illustrating that the ultimate biological effect of estrogen in the corneal endothelium is critically dependent on the specific pathophysiological environment.

This principle of context-dependency is especially pertinent to the regulation of ECM production by E2, which is a central theme of our study. In some biological settings, E2 is profibrotic; for instance, in dermal fibroblasts, E2 promotes fibrosis by inducing TGF-β1 and TGF-β2 expression,^35^ and in breast cancer cells, E2 increases syndecan-2 and MMP-9 expression through ERα signaling.^36^ In others, however, its effects are powerfully antifibrotic. E2 can suppress ECM production in breast cancer cells through GPR30-mediated inhibition of MAPK and Smad signaling,^37^ in diabetic nephropathy models by modulating TGF-β downstream signaling and suppressing type I and IV collagen accumulation,^38^ and in dermal fibroblasts by inhibiting TGF-β-dependent activation and collagen production through decreased Smad2/3 phosphorylation.^14^ Collectively, these disparate examples underscore the dual and context-dependent role of E2 in ECM homeostasis. This very principle lends strong plausibility to its potential antifibrotic action in FECD, a disease fundamentally driven by aberrant TGF-β signaling and excessive ECM production.

Our study now demonstrates that, in FECD, E2 suppresses excessive ECM production through inhibition of Smad signaling, a mechanism consistent with its established antifibrotic actions in other estrogen-responsive tissues like the breast and kidneys.^39^^,^^40^ A key consequence of this pathway’s inhibition is the downregulation of the EMT-related transcription factors *Snail1* and *ZEB1*. This finding is particularly impactful, as our previous work established these very factors as direct drivers of excessive ECM production in FECD.^25^ The present study thus elucidates a clear protective cascade: E2’s inhibition of TGF-β-induced Smad phosphorylation suppresses the core EMT program, which in turn ameliorates pathological ECM remodeling and the consequent accumulation of unfolded proteins. Beyond these molecular effects, a sex-stratified analysis revealed that E2 exerts comparable protective effects in both male and female mice. This finding is particularly insightful, as the clinical observation that FECD progression is more pronounced in postmenopausal women has long suggested a link to age-related hormonal changes. This interpretation is further supported by a recent large-scale genetic study, which reported that the female predominance in FECD is especially marked in CTG18.1 expansion-negative patients—those lacking known genetic drivers—indicating a potentially greater role for sex-specific factors, including estrogen signaling, in this subgroup.^41^ Crucially, whereas this hormonal shift is most dramatic in women, an age-related decline in estrogen also occurs in men; this provides a plausible biological basis for our key finding that E2 exerts a protective effect across both sexes. This indicates that estrogen signaling is fundamental to corneal endothelial homeostasis beyond female-specific hormonal dynamics, strongly supporting the clinical relevance of E2-based therapy for a wider patient population.

Corneal endothelial cell death in FECD has been attributed to multiple mechanisms, including endoplasmic reticulum (ER) stress from unfolded proteins, oxidative stress, RNA toxicity, TCF4 dysregulation due to TCF4 triplet repeat expansion, and ferroptosis.^1^^–^^3^ These mechanisms are believed to coexist and contribute synergistically to FECD pathogenesis.^1^^–^^3^ The central role of protein misfolding and ER stress in triggering endothelial cell loss makes the mitigation of these upstream stressors a particularly compelling therapeutic strategy. In our FECD mouse model, E2 treatment not only suppressed ECM production but also maintained a higher cell density by preventing cell death. This mouse model has been previously validated to exhibit ER stress-induced cell death^19^ that mimics the situation observed in human patients with FECD.^27^^,^^28^^,^^42^ Thus, our findings suggest a unifying protective mechanism: the E2-mediated attenuation of ECM dysregulation likely alleviates cellular proteostatic burden, a process that could plausibly reduce ER stress and thereby explain the observed preservation of endothelial cells. Whereas our study demonstrates the protective effects of E2 on early FECD phenotypes, several limitations warrant discussion. The main limitation lies in the animal model: although *Col8a2*^Q455K/Q455K^ mice faithfully replicate early pathological features such as guttae formation and cell loss, they do not progress to the corneal edema that characterizes advanced human FECD. Nevertheless, this limitation does not compromise the translational value of our findings. Corneal edema represents a late manifestation of chronic endothelial decompensation, whereas the disease process begins much earlier with guttae formation and progressive ECM accumulation. These early alterations are clinically meaningful, as guttae induce light scattering and visual impairment well before stromal swelling occurs. Therefore, by showing that E2 suppresses guttae formation and excessive ECM deposition, our study identifies a mechanism that is directly relevant to the early, vision-compromising stage of FECD. Moreover, because aberrant ECM accumulation and chronic ER stress are mechanistically linked to endothelial degeneration,^27^^,^^28^ it is conceivable that E2-mediated modulation of these early events could delay or even reduce the risk of later corneal edema, although this hypothesis requires longitudinal validation. Consequently, although our data provide strong evidence for E2’s role in mitigating upstream pathogenic events, its effects on endothelial function and tissue transparency in advanced disease stages remain to be determined. Elucidating these later-stage effects will require studies using alternative animal models or, ultimately, human clinical trials. A second limitation is that the proposed mechanism—inhibition of the TGF-β/Smad signaling axis—awaits direct confirmation in vivo, as it was established here through in vitro experiments. Fully validating the physiological relevance of our mechanistic findings will necessitate future targeted analyses of TGF-β downstream effectors, EMT markers, and proteostasis regulators in corneal tissue from E2-treated animals. Finally, although the effects of E2 were generally consistent across iFECD (RE+) and iFECD (RE−) cells, the potential influence of TCF4 repeat expansion status on E2 responsiveness remains to be fully elucidated and warrants further investigation.

The therapeutic potential of E2 is supported by its established clinical applications in various conditions that affect both men and women, including postmenopausal symptoms and postpartum depression.^43^^–^^45^ In ophthalmology, local administration through eye drops represents a particularly promising approach, as this simple application could potentially minimize systemic side effects while maintaining therapeutic efficacy. Although corneal endothelial delivery of E2 has not yet been empirically demonstrated, formulation studies—such as the development of in situ gel‑forming estradiol eye drop systems—suggest that local ocular delivery of E2 is pharmacologically feasible.^46^ Notably, a recent epidemiological study by Millen et al. demonstrated that current hormone therapy use was associated with a decreased risk of FECD in postmenopausal women, although the duration of hormone therapy use, the estimated lifetime exposure to endogenous estrogen, and the serum E2 concentrations did not show significant associations.^13^ The complexity of these epidemiological findings underscores the critical need for mechanistic studies, such as ours, that can elucidate local, tissue-specific pathways. The convergence of this epidemiological evidence with our experimental data thus provides a compelling rationale for exploring hormone-based therapies, particularly those targeting the TGF-β signaling and ECM dysregulation central to FECD.

In conclusion, our findings advance the understanding of FECD pathogenesis by identifying a protective role for E2 in modulating TGF-β–Smad signaling and ECM remodeling. Critically, this work also provides the first direct experimental evidence to support E2-based treatments. Given its established safety profile and the availability of clinically approved formulations, E2 supplementation via appropriate systemic or local routes represents a feasible and mechanistically grounded therapeutic strategy for patients with FECD.

## Supplementary Material

Supplement 1

Supplement 2
