# Supplementary material for: Protective Effects of Estradiol on Disease Progression in a Murine Model of Fuchs Endothelial Corneal Dystrophy
Source: Invest Ophthalmol Vis Sci. 2025 Dec 22;66(15):64. doi: 10.1167/iovs.66.15.64 (PMC12742592; doi:10.1167/iovs.66.15.64)
Supplement: Supplement 1 [file iovs-66-15-64_s001.pdf]

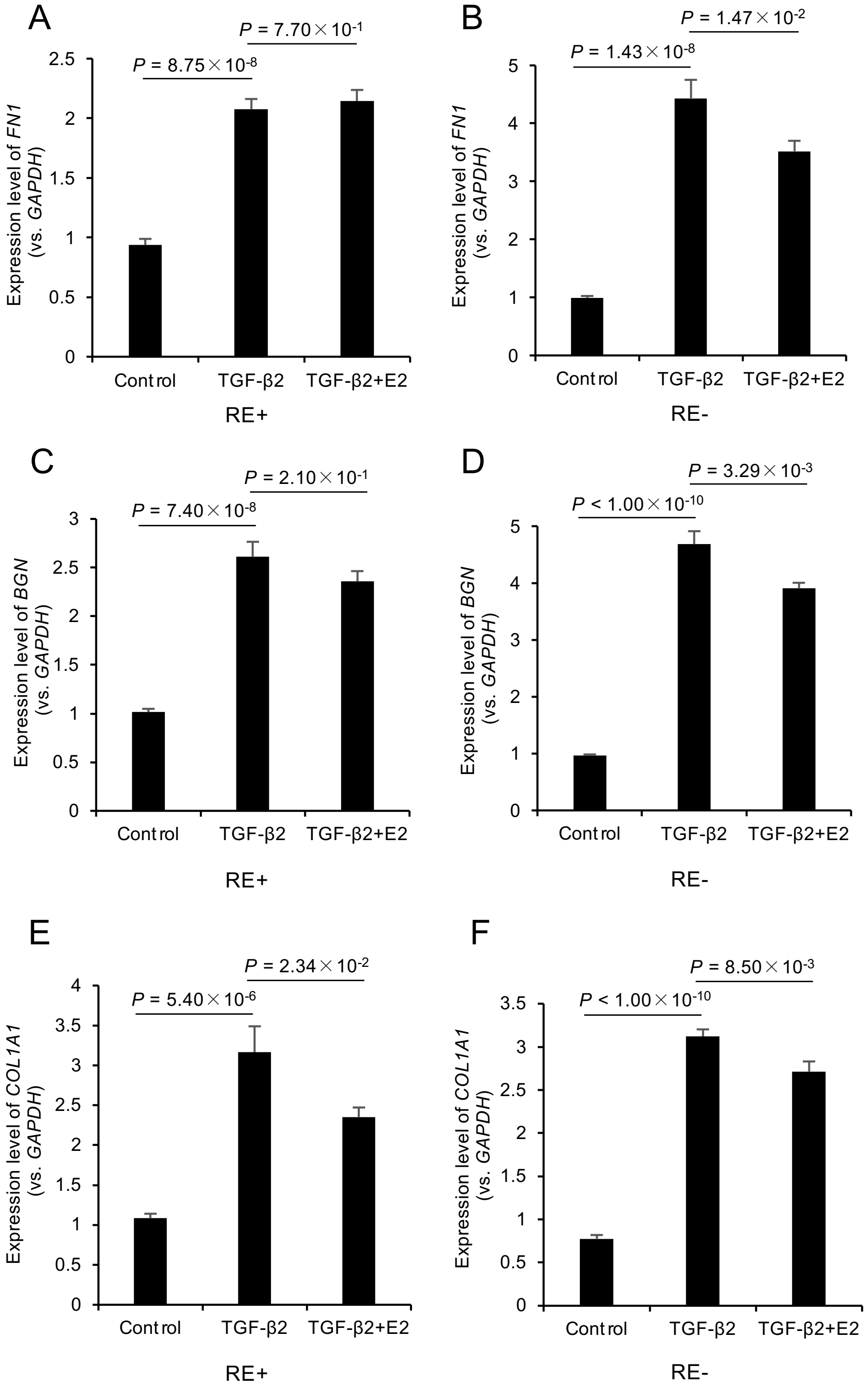

Supplementary Figure 1. Effects of Estradiol (E2) on ECM-related gene expression

Quantitative PCR analysis was performed to examine the effects of E2 on ECM-related gene expression in both iFECD (RE-) and iFECD (RE+) cells. TGF-β2 treatment significantly upregulated the expression of *FN1*, *BGN*, and *COL1A1* in both cell lines compared to controls. In iFECD (RE+) cells, co-treatment with 1 μM E2 showed no significant effect on *FN1* (A) and *BGN* (C) expression but significantly suppressed the TGF-β2-induced upregulation of *COL1A1* (E). In iFECD (RE-) cells, E2 co-treatment significantly inhibited the TGF-β2-induced upregulation of all three genes: *FN1* (B), *BGN* (D), and *COL1A1* (F).
