# Supplementary material for: Protective Effects of Estradiol on Disease Progression in a Murine Model of Fuchs Endothelial Corneal Dystrophy
Source: Invest Ophthalmol Vis Sci. 2025 Dec 22;66(15):64. doi: 10.1167/iovs.66.15.64 (PMC12742592; doi:10.1167/iovs.66.15.64)
Supplement: Supplement 2 [file iovs-66-15-64_s002.pdf]

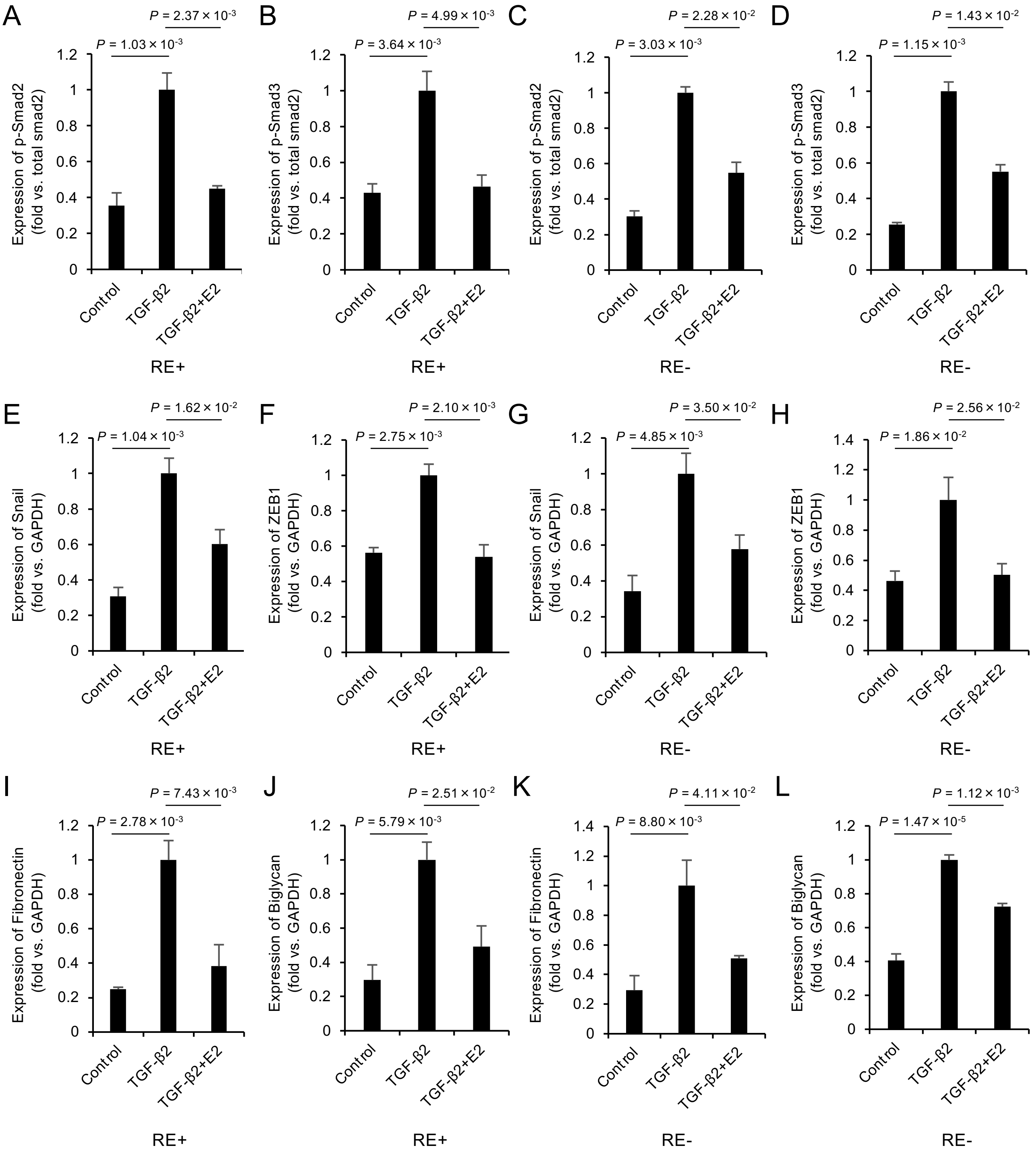

Supplementary Figure 2. Densitometric analysis of TGF-β signaling pathway proteins and their modulation by estradiol (E2) in FECD cells

(A-D) Quantification of phosphorylated Smad2 and Smad3 relative to total Smad2 expression in iFECD (RE+) (A,B) and iFECD (RE-) (C,D) cells. E2 co-treatment significantly reduced TGF-β2-induced phosphorylation of both Smad proteins in both cell lines. (E-H) Quantification of EMT-related factors Snail and ZEB1 in iFECD (RE+) (E,F) and iFECD (RE-) (G,H) cells. E2 co-treatment significantly suppressed TGF-β2-induced upregulation of both transcription factors. (I-L) Quantification of ECM proteins fibronectin and biglycan in iFECD (RE+) (I,J) and iFECD (RE-) (K,L) cells. E2 significantly inhibited TGF-β2-induced expression of these ECM components in both cell lines. P values are indicated for comparisons between control vs. TGF-β2 and TGF-β2 vs. TGF-β2+E2 groups. Western blot results were quantified by densitometric analysis using Image J software. Experiments were performed in triplicate, and data are presented as mean ± SD of relative protein expression normalized to GAPDH. Statistical significance was determined using one-way ANOVA followed by Tukey's post-hoc test.
